# Deformation and Plateau Region of Functionally Graded Aluminum Foam by Amount Combinations of Added Blowing Agent

**DOI:** 10.3390/ma8105366

**Published:** 2015-10-21

**Authors:** Yoshihiko Hangai, Takao Utsunomiya, Osamu Kuwazuru, Soichiro Kitahara, Nobuhiro Yoshikawa

**Affiliations:** 1 Graduate School of Science and Technology, Gunma University, Kiryuu 376-8515, Japan; 2 Faculty of Engineering, Shibaura Institute of Technology, Tokyo 135-8548, Japan; utunomiy@sic.shibaura-it.ac.jp; 3 Graduate School of Engineering, University of Fukui, Fukui 910-8507, Japan; kuwa@u-fukui.ac.jp; 4 Hokudai Co., Ltd., Abira 059-1434, Japan; soichiro_kitahara@hokudai-jp.com; 5 Institute of Industrial Science, the University of Tokyo, Tokyo 153-8505, Japan; yoshi@telu.iis.u-tokyo.ac.jp

**Keywords:** cellular materials, functionally graded materials, friction stir processing, pore structure, porosity, aluminum alloy die casting, foam

## Abstract

Recently, to further improve the performance of aluminum foam, functionally graded (FG) aluminum foams, whose pore structure varies with their position, have been developed. In this study, three types of FG aluminum foam of aluminum alloy die casting ADC12 with combinations of two different amounts of added blowing agent titanium(II) hydride (TiH_2_) powder were fabricated by a friction stir welding (FSW) route precursor foaming method. The combinations of 1.0–0 mass %, 0.4–0 mass %, and 0.2–0 mass % TiH_2_ were selected as the amounts of TiH_2_ relative to the mass of the volume stirred by FSW. The static compression tests of the fabricated FG aluminum foams were carried out. The deformation and fracture of FG aluminum foams fundamentally started in the high-porosity (with TiH_2_ addition) layer and shifted to the low-porosity (without TiH_2_ addition) layer. The first and second plateau regions in the relationship between compressive stress and strain independently appeared with the occurrence of deformations and fractures in the high- and low-porosity layers. It was shown that FG aluminum foams, whose plateau region varies in steps by the combination of amounts of added TiH_2_ (*i.e.*, the combination of pore structures), can be fabricated.

## 1. Introduction

Aluminum foam has various advantages, such as very light weight and good energy and vibration absorption properties, and is expected to be used as a multifunctional material practically in various industrial fields [1,2,3,4]. Recently, to further improve the performance of aluminum foam, functionally graded (FG) aluminum foams, which consist of several foam layers with different pore structures (*i.e.*, with different porosities and pore sizes), have been developed in various studies [5,6,7,8,9,10]. However, these studies mainly dealt with the methods of fabricating FG aluminum foams and there are few studies on the compressive properties of FG aluminum foams.

The authors proposed a fabrication method called the friction stir welding (FSW) route precursor foaming method for fabricating uniform aluminum foams [11,12]. Moreover, we succeeded in fabricating seamless FG aluminum foams by bonding several foam layers with different pore structures and alloy compositions [13,14,15,16]. In addition, it was shown that the compressive properties corresponding to each foam layer appeared independently in the compression tests of FG aluminum foams [14,15].

In this study, using aluminum alloy die casting ADC12 as a starting material, we attempted to fabricate three types of FG aluminum foam with combinations of two different pore structures. FG aluminum foams were fabricated using the FSW route precursor foaming method. In these fabrications, the combinations of 1.0–0 mass %, 0.4–0 mass %, and 0.2–0 mass % were selected as the amounts of titanium(II) hydride (TiH_2_) powder relative to the mass of the volume stirred by FSW, and the pore structure in each FG aluminum foam layer was distinguished. The fabricated FG aluminum foams were observed by X-ray computed tomography (CT), and the distributions of porosities and pore sizes at certain locations in the FG aluminum foams were evaluated. Subsequently, the compression tests of the FG aluminum foams were carried out, and the deformation and fracture behaviors and the plateau region for each foam layer were examined. From these examinations, the possibility of fabricating FG aluminum foams, whose plateau region varies in steps by the combination of amounts of added TiH_2_ (*i.e.*, the combination of pore structures), was discussed.

## 2. Results and Discussion

### 2.1. Distribution of Pore Structures

**Figure 1:**
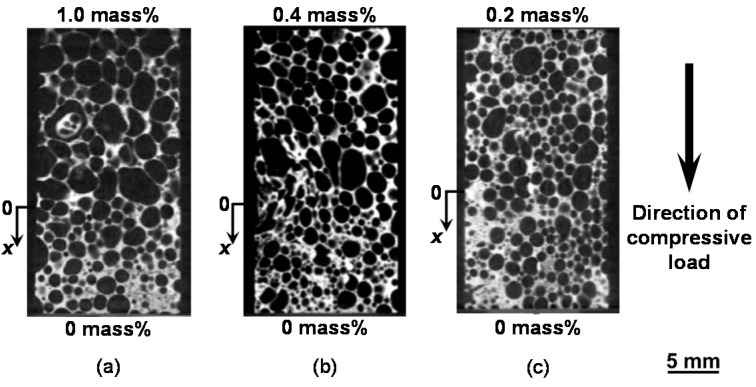
Pore structures of functionally graded (FG) aluminum foams with combinations of (**a**) 1.0–0 mass % TiH_2_; (**b**) 0.4–0 mass % TiH_2_; and (**c**) 0.2–0 mass % TiH_2_.

Figure 1a–c show the pore structures of compression specimens with the combinations of 1.0–0 mass %, 0.4–0 mass %, and 0.2–0 mass % TiH_2_ obtained from the X-ray CT images, respectively. In these figures, the direction of the compressive load in the compression test is also shown. The average densities of the specimens with the combinations of 1.0–0 mass %, 0.4–0 mass %, and 0.2–0 mass % TiH_2_ are 0.49, 0.61 and 0.68 Mg/m^3^.

**Figure 2:**
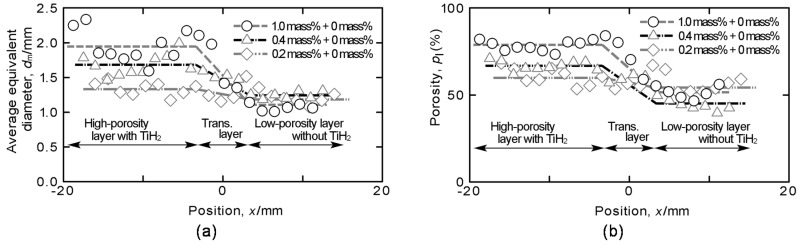
Distributions of (**a**) average equivalent diameter and (**b**) porosity on each cross-section of FG aluminum foams with three combinations of amounts of TiH_2_.

Figure 2a,b show the distributions of the average equivalent diameter *d*_m_ and porosity *p*_l_ on each cross-section perpendicular to the height directions of compression specimens with three combinations of amounts of TiH_2_, respectively. In Figure 2, the location *x* in each height direction is shown as the distance from the center position of the stirred region obtained by FSW. Here, the location of *x* = 0 slightly differs in each specimen because the stirred regions with large TiH_2_ amounts, such as 1.0 and 0.4 mass % TiH_2_, expand greatly with foaming and it is difficult to set up the center position of the stirred region precisely when the specimens are cut from the foamed precursors. In these figures, it can be seen that, although the variations caused by foaming are large, there exist a high-porosity layer with TiH_2_ (large pore size) and a low-porosity layer without TiH_2_(small pore size), and the values of *d*_m_ and *p*_l_ vary gradually in the transition layer between them [17]. Moreover, the values of *d*_m_ and *p*_l_ in the high-porosity (large-pore-size) layer decrease with decreasing amount of TiH_2_. Here, in the combination of 0.2–0 mass % TiH_2_, the differences of the two layers of *d*_m_ and *p*_l_ are small at values of approximately 6% and 0.2 mm, respectively. In particular, the difference of *d*_m_ is not as large as the variation in *d*_m_ with foaming and large pores of approximately *d*_m_ = 1.4 mm with almost the same size as those in the high-porosity layer can also be seen in the low-porosity layer.

### 2.2. Relationship between Compressive Stress and Strain

**Figure 3:**
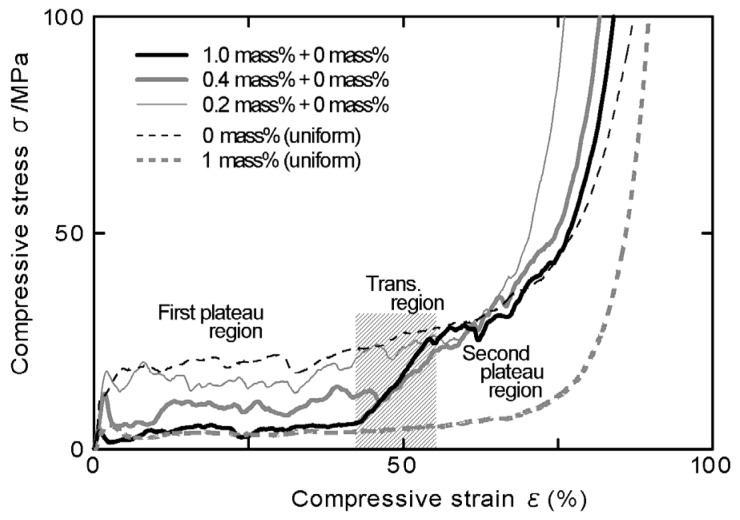
Relationship between compressive stress and strain of FG aluminum foam.

**Figure 4:**
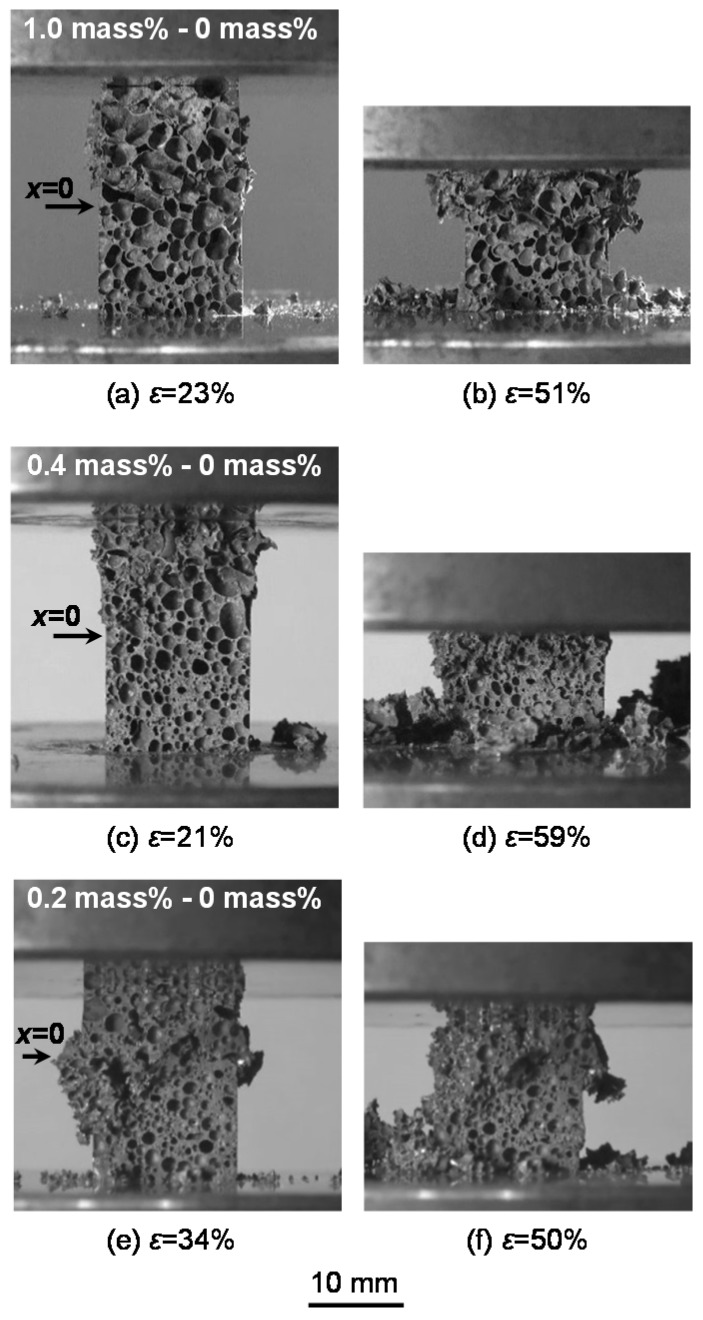
Typical fracture states: (**a**) 1.0–0 mass % TiH_2_; ε = 23%; (**b**) 1.0–0 mass % TiH_2_; ε = 51%; (**c**) 0.4–0 mass % TiH_2_; ε = 21%; (**d**) 0.4–0 mass % TiH_2_; ε = 59%; (**e**) 0.2–0 mass % TiH_2_; ε = 34%; (**f**) 0.2–0 mass % TiH_2_; ε = 50%.

Figure 3 shows the relationships between compressive stress and strain obtained from the compression test of the specimens with three combinations of amounts of TiH_2_. In this figure, the relationships for uniform foams with (1.0 mass % TiH_2_) [18] and without TiH_2_ (0 mass % TiH_2_) [19] are also shown. Figure 4a–f show typical fracture states under the compressive load of the specimens with three combinations of amounts of TiH_2_. In these figures, the arrows correspond to the location of *x* = 0 in the specimen. As shown in Figure 4a,c, the specimens with the combinations of 1.0–0 mass % and 0.4–0 mass % TiH_2_ first started to deform and fracture only in the high-porosity layers and a negligible level of deformation could be seen in the low-porosity layers. With the occurrence of deformations and fractures in the high-porosity layers, the initial maximum stresses and first plateau regions appeared as shown in Figure 3. The initial maximum stresses and the stresses in the first plateau regions increased with the decreasing amount of TiH_2_ (*i.e.*, decreasing porosity and pore size). Moreover, the stresses in the first plateau region of the specimen with the combination of 1.0–0 mass % TiH_2_ were almost the same as the plateau stress for the uniform foam with 1.0 mass % TiH_2_. The transition layers between two layers started to deform and fracture with the densification of the high-porosity layer as shown in Figure 4b,d, and the compressive stresses also started to increase. The extent of the transition region (from ε = 40% to ε = 55%) is shown in Figure 3. In this transition region, the transition layers shown in Figure 2a,b almost deformed and fractured. Also, it is considered that the end point of the first plateau region is the initial point for the densification of the high-porosity layer. Thereafter, the low-porosity layers started to deform and fracture and the second plateau regions appeared. The stresses in the second plateau regions of the specimens with the combinations of 1.0–0 mass % and 0.4–0 mass % TiH_2_ were almost the same as the plateau stress for uniform foam without TiH_2_ (0 mass % TiH_2_). Thus, by fabricating the high- and low-porosity layers intentionally, the deformation layer can be controlled in FG aluminum foam.

After the second plateau region, the densification of the low-porosity layer started with the densifications of the high-porosity and transition layers. It is considered that the point for approximately ε = 70% was the initial point for the densification of the second plateau region. Also, in FG aluminum foam, two amounts of energy absorption for the first and second plateau regions can be defined because the stresses in the first and second plateau regions were almost the same as those for uniform foams with 0 and 1.0 mass % TiH_2_, respectively.

The specimen with the combination of 0.2–0 mass % TiH_2_ fundamentally started to deform and fracture in the high-porosity layer and shifted to the deformations and fractures in the transition and low-porosity layers (*cf.* Figure 4e,f). However, as shown in Figure 4f, in addition to the deformation and fracture in the high-porosity layer, those of the local portions with large pore sizes, which are almost the same as the pore sizes in the high-porosity layer, in the low-porosity layer might occur simultaneously. Thus, in the specimen with the combination of 0.2–0 mass % TiH_2_, it is difficult to distinguish the fracture location clearly because the porosity and pore size differences of the high- and low-porosity layers become small. On the other hand, in Figure 3, we can see the first plateau region whose stress is slightly lower than the plateau stress for a uniform foam without TiH_2_, owing to the effect of the high-porosity layer with 0.2 mass % TiH_2_, and this plateau region transformed into the second plateau region gradually.

**Figure 5:**
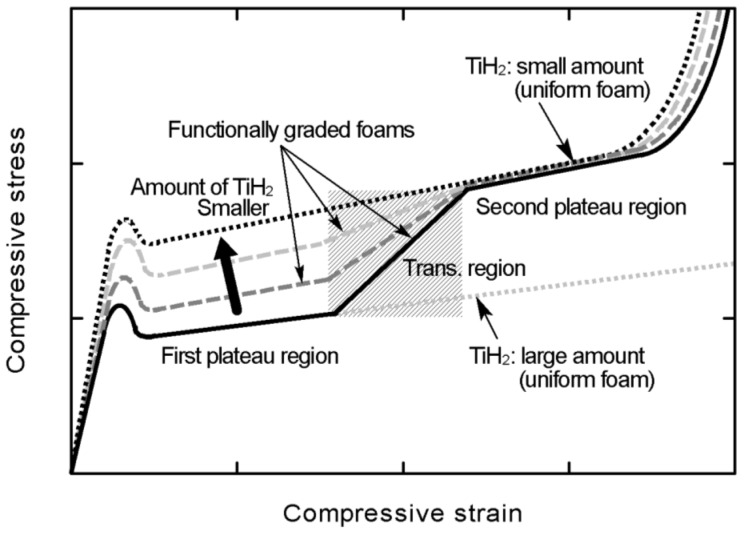
Schematic illustration of states of plateau regions varied by combination of amounts of added TiH_2_.

From the above results, the varying states of plateau regions by the combination of amounts of added TiH_2_ (*i.e.*, the combination of pore structures) of FG aluminum foams fabricated in this study are indicated in Figure 5 schematically and summarized as follows:

(1) The first and second plateau regions independently appear with the occurrence of deformations and fractures in the high- and low- porosity layers.

(2) The first plateau region gradually transformed into the second plateau region with deformation and fracture in the transition layer.

(3) The stresses in the first and second plateau regions are almost the same as the plateau stresses for uniform foams with the same amount of added TiH_2_.

Thus, FG aluminum foams whose plateau region varies in steps by the combination of amounts of added TiH_2_ (*i.e.*, the combination of pore structures) can be fabricated using the FSW route precursor foaming method.

## 3. Experimental Procedures

### 3.1. Fabrication of FG aluminum Foams

**Figure 6:**
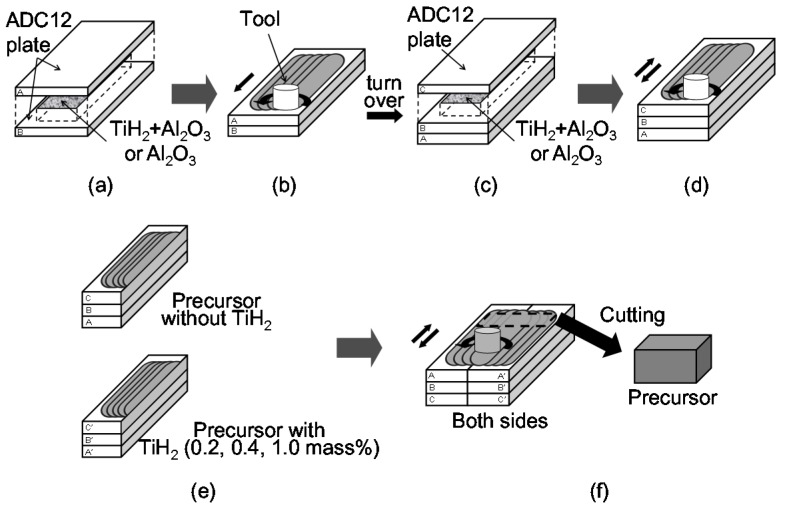
Schematic illustration of fabrication process used for functionally graded aluminum foam by friction stir welding (FSW) route precursor foaming method. (**a**) The powders were placed along the path of the FSW tool; (**b**) FSW was conducted; (**c**) The powders were placed along the path of the FSW tool; (**d**) FSW was conducted; (**e**) Precursors were cut; (**f**) Precursors were butt welded by FSW.

As starting materials, Al-Si-Cu aluminum alloy ADC12 die casting plates of 3 mm thickness, 70 mm width, and 130 mm length were used. These plates contained some gases owing to the die casting process [17,20]. The total amount of gases contained in these plates was 250.6 cm^3^/100g Al. Figure 6 shows a schematic illustration of the fabrication process used for the FG aluminum foam precursor. First, as shown in Figure 6a, two die casting plates were stacked with agent powders between them. Titanium(II) hydride (TiH_2_) powder (<45 μm) as a blowing agent and alumina (Al_2_O_3_) powder (~1 μm) as a stabilization agent were both used for one of the laminated plates and only Al_2_O_3_ powder was used for the other laminated plate. Next, the multipass FSW [12,21,22,23] of 5 lines × 4 passes, as shown in Figure 6b, was applied to obtain a larger precursor and to mix the gas and powders thoroughly. FSW was carried out using an FSW machine (SHH204-720, Hitachi Setsubi Engineering Co., Ltd., Hitachi, Japan). The amount of added TiH_2_ was selected as follows. That is, it was shown in the previous study that, when the amount of TiH_2_ varied from 0 to 0.6 mass % for ADC12 die casting, the porosity and pore structure varied markedly with the increasing amount of TiH_2_ and, when the amount of TiH_2_ was 0.6 mass % or larger, the porosities were almost the same and large pores with diameters of approximately 2 mm were formed [17]. From this finding, the values of 0, 0.2, 0.4 and 1.0 mass % were selected as the amounts of TiH_2_ relative to the mass of the die casting with dimensions of 110 mm × 30 mm × 5 mm (*i.e.*, the volume stirred by FSW) in this study. The amount of Al_2_O_3_ powder was 5 mass % relative to the mass of the aluminum with the above dimensions. Next, as shown in Figure 6c,d, the stirred plate was turned over, one more die casting plate was stacked with the same agent powders as those shown in Figure 6b on the reverse side of the area of FSW applied in Figure 6b, and the same multipass FSW as that shown in Figure 6b was applied once again to obtain a thicker precursor. Then, as shown in Figure 6e, two stirred plates without TiH_2_ (0 mass %) and with TiH_2_ of 0.2, 0.4 or 1.0 mass % were cut in the stirred region and butt-welded by FSW of 1 line × 2 passes on both sides. A precursor with dimensions of 30 mm × 50 mm × 9 mm was cut from the region including the bonding layer as shown in Figure 6f. The precursor sample was heated in a preheated electric furnace. The holding temperature (equal to the preheated temperature) and holding time during heating were 948 K and 14 min, respectively, with reference to a previous study [17]. After heating, the foamed precursor was water-cooled and the compression specimens of 30 mm height, 15 mm width, and 15 mm depth were cut by electrodischarge machining. Three specimens for each combination of 1.0–0 mass %, 0.4–0 mass %, and 0.2–0 mass % TiH_2_ are used in the experiments.

### 3.2. Evaluation of Pore Structure

The compression specimens were scanned using a microfocus X-ray CT system (SMX-225CT, Shimadzu Corporation, Kyoto, Japan). The X-ray source used in this system was tungsten. The X-ray tube voltage and current used in the inspection were 80 kV and 30 μA, respectively. The resolution of the X-ray CT image was 512 × 512 pixels and the length of one pixel was approximately 50 μm. An appropriate threshold was set to distinguish the aluminum and pores in the following processing. By layering all the two-dimensional cross-sectional X-ray CT images perpendicular to the height direction of the compression specimens, three-dimensional (3D) images of the pore structures were created using image-based structural analysis software (ImageJ 1.46r, NIH, Bethesda, MD, USA). Moreover, binarized X-ray CT images of the pore structures for all the cross-sectional X-ray CT images were obtained. From the obtained binarized images, the porosity *p*_l_ and equivalent diameter *d* of each pore defined by the following equation on each cross-section were evaluated as:

(1) $$d=2{\left(\frac{A}{\text{π}}\right)}^{\frac{1}{2}}$$

where *A* is the area of each pore. In the evaluations, pores with areas of less than 0.3 mm^2^ were excluded owing to the resolution of the X-ray CT images. Also, the average equivalent diameter *d*_m_ was calculated as the average *d* value on each cross-section. This image processing was carried out using image-processing software (WinROOF, Mitani Corporation, Fukui, Japan).

### 3.3. Compression Test

The compression test was carried out at room temperature using an Instron-type testing machine with a load capacity of 98 kN (AG-100kN, Shimadzu Corporation, Kyoto, Japan) according to Japanese Industrial Standard JIS H 7902 [24]. However, as the dimensions of the specimen of FG aluminum foam were not defined in JIS H 7902, we used the original specimen of FG aluminum foam. The relative velocity between the cross head and the screw rod was set at 6 mm/min. From the relationship between the compression load *P* and the displacement of the cross head measured in the compression test, the height change of the specimen Δ*h* was evaluated. The compression stress σ and strain ε were calculated as:

(2) $$\text{σ}=\frac{P}{A_{\text{n}}},\;\text{ε}=\frac{\text{Δ}h}{h}\times 100$$

where *A*_n_ is the cross-section of the specimen before the compression test (*A*_n_ = 15 × 15 mm^2^) and *h* is the height of the specimen before the compression test (*h* = 30 mm).

## 4. Conclusions

In this study, using the FSW route precursor foaming method, FG aluminum foams of aluminum alloy die casting ADC12 with the three combinations of 1.0–0 mass %, 0.4–0 mass %, and 0.2–0 mass % TiH_2_ were fabricated. Moreover, the compression tests of the fabricated FG aluminum foams were carried out. The experimental results led to the following conclusions:

(1) Although, in the specimen with the combination of 0.2–0 mass % TiH_2_, it is difficult to clearly distinguish the fracture location clearly because the porosity and pore size differences of two layers become small, the deformation and fracture of FG aluminum foams fundamentally started in the high-porosity (1.0, 0.4 and 0.2 mass % TiH_2_) layer and shifted to the low-porosity (0 mass % TiH_2_) layer.

(2) The first and second plateau regions independently appear with the occurrence of deformations and fractures in the high- and low-porosity layers.

(3) The initial maximum stresses and the stresses of plateau regions varied in steps with the combination of amounts of added TiH_2_ and pore structures.

(4) By fabricating the high- and low-porosity layers intentionally, the deformation layer can be controlled in FG aluminum foams.
